# Fullerene Derivatives Prevent Packaging of Viral Genomic RNA into HIV-1 Particles by Binding Nucleocapsid Protein

**DOI:** 10.3390/v13122451

**Published:** 2021-12-06

**Authors:** Ivana Křížová, Alžběta Dostálková, Edison Castro, Jan Prchal, Romana Hadravová, Filip Kaufman, Richard Hrabal, Tomáš Ruml, Manuel Llano, Luis Echegoyen, Michaela Rumlová

**Affiliations:** 1 Department of Biotechnology, University of Chemistry and Technology Prague, Technická 5, 166 28 Prague, Czech Republic; krizovaa@vscht.cz (I.K.); dostalkl@vscht.cz (A.D.); romana.hadravova@uochb.cas.cz (R.H.); kaufmanf@vscht.cz (F.K.); 2 Department of Chemistry, University of Texas at El Paso, 500 West University, El Paso, TX 79902, USA; edisoncastro2004@hotmail.com (E.C.); echegoyen@utep.edu (L.E.); 3 Laboratory of NMR Spectroscopy, University of Chemistry and Technology, 166 28 Prague, Czech Republic; prchalj@vscht.cz (J.P.); hrabalr@vscht.cz (R.H.); 4 Department of Biochemistry and Microbiology, University of Chemistry and Technology Prague, Technická 5, 166 28 Prague, Czech Republic; rumlt@vscht.cz; 5 Department of Biological Sciences, University of Texas at El Paso, 500 West University, El Paso, TX 79902, USA; mllano@utep.edu

**Keywords:** HIV-1, fullerene, nucleocapsid, inhibition, RNA packaging

## Abstract

Fullerene derivatives with hydrophilic substituents have been shown to exhibit a range of biological activities, including antiviral ones. For a long time, the anti-HIV activity of fullerene derivatives was believed to be due to their binding into the hydrophobic pocket of HIV-1 protease, thereby blocking its activity. Recent work, however, brought new evidence of a novel, protease-independent mechanism of fullerene derivatives’ action. We studied in more detail the mechanism of the anti-HIV-1 activity of *N*,*N*-dimethyl[70]fulleropyrrolidinium iodide fullerene derivatives. By using a combination of *in vitro* and cell-based approaches, we showed that these C_70_ derivatives inhibited neither HIV-1 protease nor HIV-1 maturation. Instead, our data indicate effects of fullerene C_70_ derivatives on viral genomic RNA packaging and HIV-1 cDNA synthesis during reverse transcription—without impairing reverse transcriptase activity though. Molecularly, this could be explained by a strong binding affinity of these fullerene derivatives to HIV-1 nucleocapsid domain, preventing its proper interaction with viral genomic RNA, thereby blocking reverse transcription and HIV-1 infectivity. Moreover, the fullerene derivatives’ oxidative activity and fluorescence quenching, which could be one of the reasons for the inconsistency among reported anti-HIV-1 mechanisms, are discussed herein.

## 1. Introduction

During its replication cycle, HIV-1 forms two distinct particles, immature and mature ones. An immature HIV-1 particle is formed by Gag polyprotein precursors consisting of four structural domains—matrix (MA, p17), capsid (CA, p24), nucleocapsid (NC), and p6—and two short spacer peptide sequences linking CA with NC (SP1) and NC with p6 (SP2). The assembly of the immature HIV-1 particle is driven mainly by interactions among CA domains of Gag polyprotein triggered by the NC–viral genomic RNA scaffold. The NC domain specifically recognizes, dimerizes, and packages viral genomic RNA into a nascent immature particle. CA-mediated multimerization on the inner leaflet of the plasma membrane (PM) leads to the formation of the hexameric lattice of the immature particle that buds into the extracellular environment. The transition to a mature, fully infectious HIV-1 particle is triggered by activation of the virus-encoded protease (PR), which cleaves Gag polyproteins into individual viral proteins. CA proteins released from the Gag precursor re-assemble to form a mature viral core. The mature HIV-1 hexameric lattice, formed approximately by 250 CA hexamers and 12 pentamers [1,2,3,4], encloses and protects viral genomic RNA, complexed to NC. The insertion of CA pentamers allows the formation of a closed conical core, characteristic of HIV-1 [2,5].

To initiate the replication cycle, HIV-1 must deliver its genome into the host cell. After HIV-1 enters the cell, the protective mature core is removed in a stepwise process called uncoating. This disassembly process, which is not well understood [6,7,8,9,10,11,12,13,14], is connected to both reverse transcription and transport toward the nucleus, where viral DNA integration into the cellular genome takes place [12,15,16]. The nucleic acid chaperone activity of NC facilitates the process of reverse transcription, during which viral genomic RNA is reversely transcribed into double-stranded DNA. The key structural and functional roles of CA and NC make them attractive pharmacological targets [17,18,19,20,21].

After diamond and graphite, fullerenes were discovered to be the third family of carbon allotropes [22]. They are highly symmetric cages of different sizes (e.g., C_60_) with unique structures and physical properties. To improve their lack of solubility, several fullerene derivatives containing hydrophilic groups have been reported [23,24]. These derivatives have useful medicinal applications, as they possess a broad spectrum of biological activities, such as antiviral, antimicrobial, antiproliferative, antioxidant, neuroprotective, and anti-cancer [24,25,26,27]. Concerning their anti-viral activities, proline-based fullerene derivatives inhibit the activity of NS3/4A protease and NS5B polymerase of the flaviviral hepatitis C virus (HCV) [23,28,29]. Giant glycofullerenes were reported to function as potent inhibitors of a reporter of Ebola virus [30,31]. Several cationic, anionic, and zwitterion fullerene derivatives of proline type, pyrrolidinium type, pyridine/pyridinium type, and others, were shown to block the activity of HIV-1 reverse transcriptase [23,28,32]. A water-soluble, (bis-(phenethylamino-succinate) C_60_ fullerene derivative was the first one reported to block the activity of HIV-1 protease *in vitro*. The mechanism of inhibition—i.e., fullerene binding to its ellipsoid-shaped, hydrophobic active site—was postulated based on in silico predictions [33,34]. Recent studies confirmed that C_60_ and C_70_ fullerene derivatives of pyrrolidinium type indeed strongly inhibit HIV-1 replication—without impairing viral protease activity, however [35,36]. Using immunoprecipitation experiments with magnetic beads functionalized with *N*,*N*-dimethyl[70]fulleropyrrolidinium iodide showed that the interactions of these C_70_ fullerene derivatives occur with immature HIV-1 CA-SP1 protein [36]. Based on this result, a model was proposed in which binding of C_70_ fullerene derivatives to the CA domain of Gag polyprotein modifies either the conformation or the assembly of the HIV-1 polyproteins, thereby altering the specificity or activity of HIV-1 PR. This model led to the conclusion that C_70_ fullerene derivatives act as the inhibitors of maturation [36].

**Figure 1:**
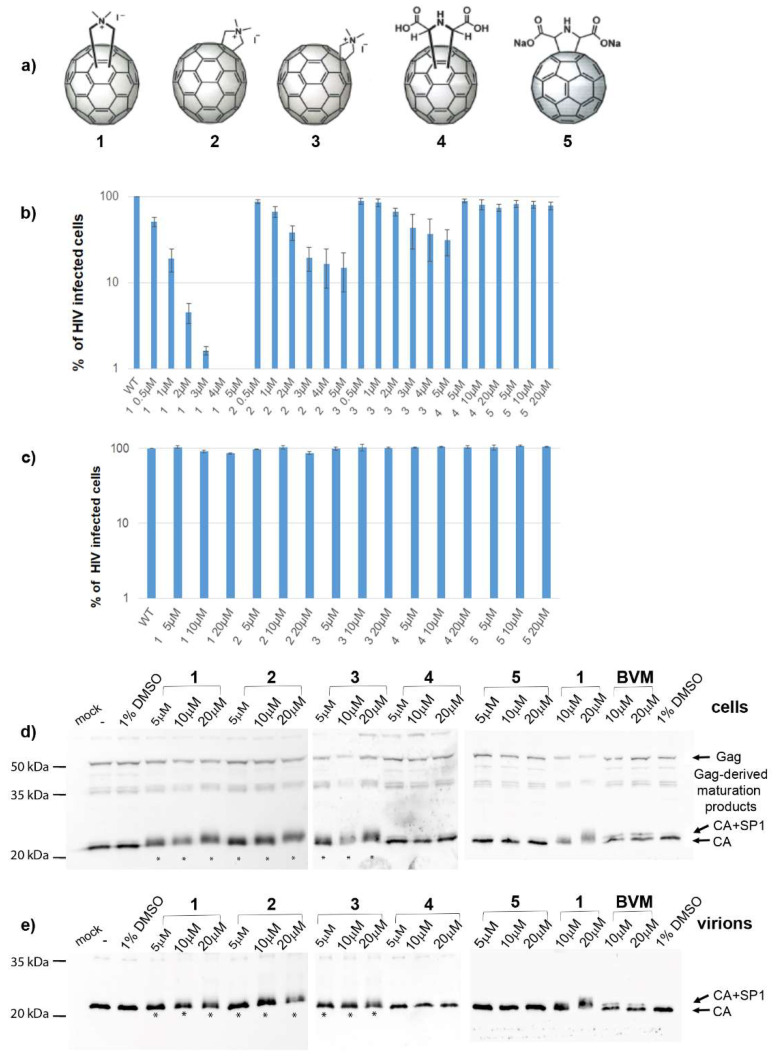
Fullerene derivatives and their effects on the HIV-1 life cycle. (**a**) Structures of fullerene derivatives **1**–**5**. (**b**) HEK 293 cells produced VSV-G-pseudotyped GFP-HIV-1 in the presence of DMSO or fullerene derivatives at indicated concentrations. At 48 h post-transfection, a normalized amount of the VSV-G pseudotyped GFP-HIV-1 released into the culture media was used for infection of fresh HEK 293 cells, and 48 h later, the GFP-positive cells were quantified using a flow cytometer. (**c**) Effects of fullerenes on the early stage of HIV-1 life cycle. ELISA-normalized amounts of VSV-G pseudotyped GFP-HIV-1 viruses were used for infection of HEK 293 cells in the presence of DMSO or indicated concentrations of fullerene derivatives. Forty-eight hours later, the GFP-positive cells were quantified using a flow cytometer. (**d**) Immunoanalysis of intracellular VSV-G pseudotyped GFP-HIV-1 virus produced in HEK 293 cells in the presence of DMSO, fullerene derivatives, or bevirimat at the indicated concentrations. (**e**) Immunoanalysis of the VSV-G pseudotyped GFP-HIV-1 virus released from the HEK 293 cells.

We tried to unveil the mechanism of action of *N*,*N*-dimethyl[70]fulleropyrrolidinium iodide fullerene derivatives (Figure 1a). In accordance with the recently published data [36], we confirmed that these fullerene derivatives had no effect on HIV-1 protease activity in either *in vitro* or cell-based experiments. Moreover, microscale thermophoresis (MST) analysis showed a low binding affinity of fullerene **1** to HIV-1 protease. Additionally, our data showed that HIV-1 Gag maturation was not affected by fullerene **1**. No effect of fullerene **1** was observed in HIV-1 RT activity either. However, in agreement with Martinez et al. [35], we observed strong inhibition of all steps of HIV-1 cDNA synthesis during reverse transcription. We correlated this observation with the finding that virions released from the cells treated with fullerene **1** contained less viral genomic RNA (gRNA); the amount of gRNA was inversely proportional to the inhibitor concentration. MST and the electrophoretic mobility shift assay (EMSA) revealed a strong binding affinity of fullerene **1** to HIV-1 NC and nucleic acids.

In conclusion, our data suggest that fullerene **1**, due to strong binding affinity for the HIV-1 nucleocapsid domain, prevents the proper interaction of viral genomic RNA with the Gag polyprotein, thereby blocking HIV-1 infectivity. We also found out fullerene **1** possesses both oxidative and fluorescence quenching activities, strongly interfering with some analysis (e.g., ELISA). We think that this feature could be one of the reasons for inconsistency among reported anti-HIV-1 mechanisms.

## 2. Materials and Methods

### 2.1. Synthesis of Fullerenes ***1**–**5***

Fullerenes **1**–**3** were synthesized by following previously reported methodologies [36,37]. Briefly, a mixture of C_70_ (84 mg, 0.1 mmol), sarcosine (22 mg, 0.24 mmol), and paraformaldehyde (18 mg, 0.60 mmol) was refluxed in toluene (75 mL) for 1 h. The resulting brown solution was concentrated in vacuo, and the crude mixture was purified by silica-gel chromatography using toluene as eluent. Pure C_60_- and C_70_-fullerene derivatives were obtained by HPLC using a Buckyprep column and toluene as eluent. Fullerenes **1**–**3** were synthesized from their corresponding neutral starting materials, which were dissolved in methyl iodide and stirred for 48 h at room temperature. The black precipitate was filtered and washed with CS_2_, CHCl_3_, toluene, MeOH, and acetone, in that order. The precursors of 4 and 5 were synthesized by following our previously reported methodology [38]. C_60_ or C_70_ (0.10 mmol) was dissolved in chlorobenzene (20 mL) under sonication for 5 min; then DIB (200 mg, 0.62 mmol), glycine methyl ester hydrochloride (100 mg, 0.79 mmol) and sodium carbonate decahydrate (100 mg, 0.35 mmol) were added. The flask was wrapped with aluminum foil and sonicated for 30 min. The pure C_60_ mono-adduct or the C_70_ mono-regioisomeric mixture was obtained after a silica gel column purification using a toluene/ethyl acetate (9:1) mixture. The corresponding dicarboxylic methyl ester C_60_/C_70_ derivative (0.015 mmol) was dissolved in toluene (20 mL) and NaH (120.00 mg, ~0.120 mmol); then, 50–60% in oil was slowly added and the reaction was stirred at room temperature for 1 h. Then, ethanol (3 mL) was added followed by the addition of HCl (3 M, 2 mL). Then, the reaction was stirred at room temperature overnight. The solvent was evaporated under reduced pressure; the precipitate was washed with chloroform, methanol, and cold water; and finally, compound **4**/**5** was recrystallized from methanol/acetone.

### 2.2. VSV-G Pseudotyped HIV-1 Particles Production

HIV-1 particles were obtained from HEK 293 cells, cotransfected by a combination of three vectors: packaging psPAX2 vector encoding HIV Gag, Pol, Tat, and Rev; reporter/transfer pWPXLd-GFP vector encoding LTR, RRE, and GFP as a reporter; and envelope pHEF-VSV-G vector, encoding vesicular stomatitis virus Env, VSV-G. The psPAX2 vector was kindly provided by Dr. Jeremy Luban; the pWPXLd-GFP and pHEF-VSV-G vectors were purchased from Addgene. HEK-293 cells were grown in Dulbecco’s modified Eagle medium (DMEM, Sigma, St. Louis, MI, USA) supplemented with 10% fetal bovine serum (Sigma, St. Louis, MI, USA) and 1% l-glutamine (Sigma, St. Louis, MI, USA) at 37 °C under 5% CO_2_. A day before transfection, cells were plated at 3 × 10^5^ cells per well. The following day, cells were transfected with the appropriate vectors using polyethylenimine (PEI, 1 mg/mL) at a 2:1 PEI:DNA ratio. Four hours post transfection, the culture medium was replaced with fresh DMEM, containing various concentrations of tested compounds, solubilized in DMSO. At 48 h post-transfection, the culture media containing released virions were harvested, filtered through 0.45-µm pores membrane, and used for immunochemical quantification and characterization by Western blot using rabbit anti-HIV-1 CA antibody.

### 2.3. Single-Round Infectivity Assay HIV-1

The infectivity was determined similarly as described earlier [39]. Briefly, 48 h post-transfection, the culture media from HEK 293 cells transfected with psPAX2, pWPXLd-GFP, and pHEF-VSV-G vectors at a ratio 1:1:1, in the presence of tested compounds, were collected and filtered through a 0.45 µm membrane filter. HIV-1 CA content was determined by ELISA. The freshly seeded HEK 293 cells were infected with WB-normalized amounts of VSV-G pseudotyped HIV-1 particles and incubated for 48 h. The cells were fixed with 2% paraformaldehyde and transferred to a FACS tube. Quantification of GFP-positive cells was performed using a BD FACS Aria III flow cytometer.

### 2.4. Flow Cytometry

The VSV-G-GFP-HIV infected cells were analyzed with a BD FACS Aria III flow cytometer (Becton Dickinson, Franklin Lakes, NJ, USA) with excitation at 488 nm and emission separated by a 530/30 band pass filter, as described earlier [39]. The obtained data were analyzed with FACSDiva software, version 8.0.

### 2.5. Transmission Electron Microscopy Analysis

HEK 293 cells producing VSV-G pseudotyped HIV-1 particles were washed with PBS 48 h post-transfection and fixed with freshly prepared 3% glutaraldehyde in 0.1 M cacodylate buffer, pH 7.5. The cells were then postfixed in 1% osmium tetroxide, dehydrated in an ethanol concentration series (30%, 50%, 70%, 80%, 90%, and 100%), and embedded in Agar 100 epoxy resin. Ultrathin sections (70 nm) prepared using an RMC MT 7000 Ultramicrotome were placed onto 200-mesh copper grids and contrasted with saturated uranyl acetate and lead citrate. The samples were then analyzed with a transmission electron microscope (JEOL; JEM-1200E×, Tokio, Japan) operating at 60 kV.

### 2.6. Quantification of VLP Assembly Efficiency by FAITH

HIV-1 CANC protein (18 µM) or ΔMACANCSP2 (15 µM) in 96-well plate was pre-incubated with tested inhibitors in the molar ratio 1:1 and kept on ice for 1 h. To start assembly reaction dually labelled oligonucleotide (tqON) was added to proteins in the molar ration protein:tqON 10:1, and the volume of the reaction mixture was adjusted to 100 µL by assembly buffer (50 mM Tris, pH 8.0, 1 µM ZnCl_2_, 340 mM NaCl). Following 3 h incubation at room temperature, Exonuclease I (ExoI) with Mg^2+^ ions was added, and the fluorescence of the fluorophore released from degraded tqON was measured using a Tecan M200Pro plate reader. The efficiency of assembly is calculated based on the formula E = 100 × ∆F2/∆F1, in which ∆F1 corresponds to the difference between relative fluorescence of tqON and relative fluorescence of tqON in the presence of HIV-1 Gag-derived protein, and ∆F2 corresponds to the difference between relative fluorescence of tqON and relative fluorescence of tqON in the presence of HIV-1 Gag-derived protein and inhibitors.

### 2.7. Western Blot and Semi-Quantitative Western Blot

At 48 h post-transfection, 100 µL aliquots of virus-containing culture media were combined with 20 µL of PLB (6×), and the samples were analyzed by Western blot using rabbit anti-HIV-1 CA (p24) (in house production). Proteins were resolved by reducing SDS-PAGE and blotted onto a nitrocellulose membrane. The antigen-antibody complexes were detected by Clarity™ Western ECL Substrate (Bio-Rad) and visualized using FUSION 7S system (Vilber Lourmat, Marne-la-Vallée, France). As CA(p24)-ELISA based normalization of HIV-1 viral particles could not be used due to interference of tested fullerene 1 with TMB reaction, semi-quantitative Western blot was used as described earlier [40]. To quantify and normalize the amount of produced HIV-1 particles, the standard curve was prepared from recombinantly expressed and purified HIV-1 CA(p24). Concentrations of CA(p24) protein from 1 to 2000 ng were separated on 15% SDS-PAGE gels and transferred onto a nitrocellulose membrane. Then, the same Western blot procedure described above was performed. The Western blot signals of CA(p24) in the calibration curve, and the viral samples, were determined using Quantum gel documentation imaging system (Vilbert Lourmat), and the amount of CA(p24) was calculated.

### 2.8. Pulse-Chase Experiment

This experiment was performed as described earlier [41,42,43] with some modifications. Forty-eight hours after transfection with appropriate DNA vectors in the presence of increasing amounts of fullerene 1, HEK 293 cells were starved for 30 min in methionine- and cysteine-deficient DMEM, pulse-labeled for 30 min with 125 Ci/mL Tran35S-label (MGP, Zlín, Czech Republic), and chased overnight in complete DMEM. The cells from pulse and pulse-chase experiments were lysed in lysis buffer (25 mM Tris-HCl pH 8.0 containing 50 mM NaCl, 1% Triton X-100, and 1% sodium deoxycholate) and centrifuged at 14,000× *g* for 2 min. The culture medium from the chased cells was filtered through a 0.45 µm membrane filter, and SDS was added to a final concentration of 0.1%. Viral proteins from both the cellular lysates and culture medium were immunoprecipitated by using an anti-HIV-1 CA antibody, followed by incubation with immobilized protein A (Invitrogen, Waltham, MA, USA). Radiolabeled proteins were separated by SDS-PAGE and detected by using a Typhoon PhosphorImager (Cytiva, Marlborough, MA, USA).

### 2.9. qPCR Analysis of Reverse Transcription Proceeding

Three different products of reverse transcription were analyzed using qPCR experiments performed according to previously reported protocol [44]. VSV-G pseudotyped HIV-1 particles produced in the absence or presence of 5 µM fullerene 1 were used to infect fresh HEK 293 cells. At various times post-infection (2, 4, 6, 10, 24, 33, and 48 h), the cells were collected, pelleted, washed with PBS, and used for the isolation of total DNA with the DNeasy blood and tissue kit (Qiagen, Hilden, Germany), according to the manufacturer’s protocol. To determine the level of different reverse transcription products, 2 µL of the isolated total DNA was mixed with qPCR 2× SYTO-9 master mix (Top-Bio, Vestec, Czech Republic), ROX as the reference dye at final concentration 25 nM, and 1 µM primers: ssDNA-F (5′-TAA CTA GGG AAC CCA CTG C-3′) and ssDNA-R (5′-GCT AGA GAT TTT CCA CAC TG-3′); EGFP-F (5′- GCG CAC CAT CTT CTT CAA GG-3′) and EGFP-R (5′-GTG TCG CCC TCG AAC TTC AC-3′); 2-LTR-F (5′-TAA CTA GGG AAC CCA CTG C-3′) and 2-LTR-R (5′-CTG CGT CGA GAG AGC TCC TCT GGT T-3′). To establish a calibration curve, appropriate DNA complementary to pairs of the primers was used in the concentration range of 0.06 to 250 pg/µL. To normalize the amounts of isolated viral DNAs among the individual samples, two endogenous control genes, encoding the phospholipase A (PLA) and C-C chemokine receptor type 5 CCR5, were selected: PLA-F (5′-AAG TTC TTG ATC CCC AAT GCT T-3′) and PLA-R (5′-GTC TGA TAG GAT GTG TTG GTT GC-3′); CCR5-F (5′-CCA GAA GAG CTG AGA CAT CCG-3′) and CCR5-R (5′-GCC AAG CAG CTG AGA GGT TAC T-3′)). qPCRs were performed in 96-well plates by using a QuantStudio 5 real-time PCR system (Applied Biosystems, Waltham, MA, USA) under the following reaction conditions: 2 min at 95 °C, followed by 45 cycles of 1 min at 95 °C, 30 s at 60 °C, and 30 s at 72 °C.

### 2.10. qPCR Analysis of Incorporated gRNA

gRNA incorporated into the HIV-1 particles was isolated and quantified as previously described [45]. Briefly, 48 h after transfection, the medium containing HIV-1 particles was harvested, filtered through 0.45 µm pores and centrifuged through 20% sucrose cushion at 210,000× *g* for 90 min in the SW41Ti rotor (Beckman Coulter, Brea, CA, USA). The pellet was resuspended in 160 µL of PBS: 140 µL was used to isolate gRNA, and the rest was analyzed using Western blot. Total RNA was isolated by using QIAamp viral RNA minikit (Qiagen, Hilden, Germany). The isolated viral RNA was incubated with Turbo DNase for 30 min at 37 °C, and the reaction was stopped by heating the mixture at 70 °C for 10 min. To control the following processes: reverse transcription and qPCR, TATAA universal RNA spike I (TATAA Biocenter, Gothenburg, Sweden) was added to each sample before reverse transcription (RT). Before RT, the amount of gRNA was normalized to the CA (p24) amount using a semi-quantitative Western blot. gRNA was transcribed to cDNA using FeLV reverse transcriptase (prepared in house). Subsequently, cDNA was used as a template in qPCR reaction prepared by mixing of 2× SYTO-9 master mix and EGFP-specific primers: EGFP-F (5′-GCG CAC CAT CTT CTT CAA GG-3′) and EGFP-R (5′-GTG TCG CCC TCG AAC TTC AC-3′). Specific primers, designed by TATAA Biocenter, were used to quantify the control RNA spikes. qPCR was performed by using a QuantStudio 5 real-time PCR system (Applied Biosystems, Waltham, MA, USA).

### 2.11. Cyclosporine Washout Assay

The cyclosporine A (CsA) washout assay in Owl monkey kidney (OMK) cells was performed as described earlier [39,46]. VSV-G pseudotyped particles were produced in HEK 293 cells in the presence or absence of CsA and with various amounts of fullerene 1. OMK cells were seeded in a 48-well plate in Eagle’s minimum essential medium (EMEM). The next day, the OMK cells were spinoculated with a normalized amount of the HIV-1 particles in EMEM containing 2.5 µM CsA and polybrene (5 ng/µL). Optionally, PF74 (5 µM) was added to OMK cells concomitantly with the infection. At indicated times, the cultivation medium containing CsA was replaced with fresh medium without CsA. After 48 h, the cells were fixed with 2% formaldehyde and GFP-positive cells were counted using flow cytometry.

### 2.12. The Activity of Reverse Transcriptase

The medium containing VSV-G pseudotyped HIV-1 particles produced in the absence or presence of fullerene 1 was harvested and filtered through 0.45 µm pores. The activity of reverse transcriptase in individual samples was then determined using the reverse transcriptase Colorimetric Assay (Sigma, St. Louis, MI, USA) according to the manufacturer’s protocol, as described earlier. The absorbance of the colorimetric reaction was measured using an Infinite^®^ 200 PRO series spectrophotometer (Tecan M200, TECAN, Männedorf, Switzerland) at 490 nm [45].

### 2.13. Thermophoresis

The HIV-1 proteins were fluorescently labeled using Protein Labeling Kit Red-NHS (Nanotemper Technologies, München, Germany). The labeling reaction was performed according to the manufacturer’s instructions. The 20 µM protein was mixed with the dye (1:3 molar) and reacted for 30 min. The unreacted dye was removed with supplied dye removal column. The labeled proteins and tubules were diluted 20×, and 5% DMSO was added. The labeled RNA and DNA oligomers were diluted to a concentration 20 nM. Fullerene **1** was dissolved in the same buffer and a series of 16 1:1 dilutions. For the measurement, each ligand dilution was mixed with one volume of labeled molecules, which led to a final concentration of fullerene ranging from 50 µM to 15 nM. The samples were loaded into Monolith NT.115 Capillaries (NanoTemper Technologies, München, Germany). MST was measured using a Monolith NT.115 instrument (NanoTemper Technologies, München, Germany) at temperature of 25 °C. Instrument parameters were adjusted to 100% LED power and medium MST power. Data of three independent measurements were analyzed using Affinity Analysis software version 2.3 (NanoTemper Technologies, München, Germany) using the signal from an MST over 1.5 s and the initial fluorescence data.

### 2.14. Electrophoretic Mobility Shift Assay (EMSA)

The binding affinity of **1** to HIV-1 CA-NC was verified and visualized using Electrophoretic Mobility Shift Assay (EMSA) according to an established protocol [14,47]. Reaction mixtures in the buffer containing 20 mM Tris-HCl, pH 7.5, 100 mM NaCl were prepared to keep the final concentrations of nucleic acid (1 kB DNA Ladder, NEB, Ipswich, MA, USA) 200 ng, and those of HIV-1 CA, NC, and CANC protein 1 μM. The final concentrations of **1** in the reactions were 1, 2, and 5 μM, corresponding to the final molar ratios of protein:fullerene **1** of 1:1, 1:2, and 1:5. To assess the binding affinity of **1** to nucleic acid, DNA was incubated with various amounts of**1** for 40 min at RT. When interactions among protein–fullerene **1**–nucleic acid were analyzed, two experimental arrangements were tested. In the first one, HIV-1 NC or CANC proteins were incubated for 20 min at RT with an excessive amount of DNA, along with fullerene **1** in different amounts**.** After incubation for 20 min at RT, the samples were resolved by 0.8% agarose gel electrophoresis. In the second arrangement, NC or CANC proteins were first preincubated for 20 min at RT with fullerene 1, and then DNA was added. Incubation proceeded for another 20 min at RT. To ensure that proteins were not contaminated with nucleic acids, the protein samples were incubated in the presence of DMSO (1%) and then loaded on the agarose gel. All samples were analyzed using 0.8% agarose gel electrophoresis in TAE (1×) buffer at 110 V, stained with Gel Red, and visualized with a Quantum gel documentation imaging system (Vilbert Lourmat, Collégien, France).

### 2.15. Docking of N,N-Dimethyl[70] Fulleropyrrolidinium Iodide

The structures of capsid (3NTE) and nucleocapsid proteins (NC) were obtained from the Brookhaven database (1F6U) by removing the stem-loop SL2 of the ψ-RNA packaging signal. The ligand structure was built from the C_70_ fullerene, downloaded from the CHEBI server (https://www.ebi.ac.uk/chebi/searchId.do?chebiId=CHEBI%3A33195, accessed on: 14 March 2021). Docking of fullerene 1 was carried out in two programs, i.e., PLANTS [48] and AutoDock Vina, version 1.5.6rc3 [49]. Both the ligand and protein were prepared correctly in auxiliary software delivered with the docking programs.

## 3. Results

### 3.1. Effects of Fullerene Derivatives on HIV-1 Infectivity

Recent work reported a novel mechanism of fullerene C_60_ and C_70_ *N*,*N*-dimethylpyrrolidinium iodide salts on HIV-1 replication [35,36]. Surprisingly, these inhibitors acted through a protease-independent mechanism, and blocked HIV-1 maturation. To further explore the mechanisms of action of these fullerenes, we synthesized and studied the effects of some cationic and anionic C_70_ fullerenes (Figure 1a, compounds **1**–**3** and **4** and **5**, respectively).

Fullerenes **1**–**5** (Figure 1a) were first tested for their cytotoxicity. None of the tested compounds revealed any cytotoxic effect in the tested concentration range (1–50 µM) on HEK 293 cells (data not shown). Next, we tested their impacts on HIV-1 via single-round infectivity assay using VSV-G-pseudotyped HIV-1 particles. The virus was produced upon transfection of HEK 293 cells with appropriate vectors in the presence of the fullerene derivatives, and the released HIV-1 particles were used for infection of fresh cells. We observed significant blocking of HIV-1 infectivity as follows: **1** (EC_50_ 1.5 µM), **2** (EC_50_ 1.8 µM), and **3** (EC_50_ 2.7 µM) (Figure 1b). Compounds **4** and **5** showed substantially lower inhibition of HIV-1 infectivity, with EC_50_ values of 37 and 27 µM, respectively. In agreement with published data [35,36], we did not observe any effect of these fullerenes on the early phase of HIV-1 infection—i.e., when these compounds were added to the HEK 293 cells infected with normalized amounts of reporter HIV-1 (Figure 1c).

The production of the virus in the HEK 293 cells and its release into culture media were monitored immunochemically using rabbit anti-HIV-CA antibody (Figure 1d,e). Gag and Gag-derived proteins were detected in the transfected cells in the presence or absence of the tested fullerenes (Figure 1d). Similarly, fully processed capsid protein was detected in the virus released into the culture media from the infected cells in the presence or absence of fullerenes (Figure 1e). Since fullerene derivatives **1**–**3** were suggested to act as maturation inhibitors [36], we compared the cleavage pattern of HIV-1 Gag-polyprotein produced in the presence of these fullerenes with that produced in the presence of bevirimat (BVM), a well-known HIV-1 maturation inhibitor. BVM blocks the final maturation step—a cleavage of p25 to p24CA and SP1 [50] (Figure 1e). In the cell lysates, Gag cleavage products, including the not fully processed p25 protein, were visible in all samples (Figure 1d). In media-associated samples (released viruses), a block of p25 to p24 cleavage was apparent only in the virus produced in the presence of BVM (Figure 1e right panel). No maturation block of p25 was observed for the samples incubated with fullerenes (Figure 1e, left panel). In agreement with Castro et al. [36], the capsid proteins in both cell-associated and media-associated samples revealed a fuzzy-like appearance of lines in the SDS-PAGE gels, which increased with the fullerene concentration in samples with fullerenes **1**–**3** (Figure 1d,e, asterisks). Such an effect was not observed with fullerenes **4** and **5**, which did not inhibit HIV-1’s infectivity. The most effective blocking of HIV-1’s infectivity was observed for fullerene 1. Since compound **1** represents an isomeric mixture of the pure isomeric forms **2** and **3**, we used only fullerene 1 for further studies.

### 3.2. Effects on HIV-1 Gag Processing and Maturation

**Figure 2:**
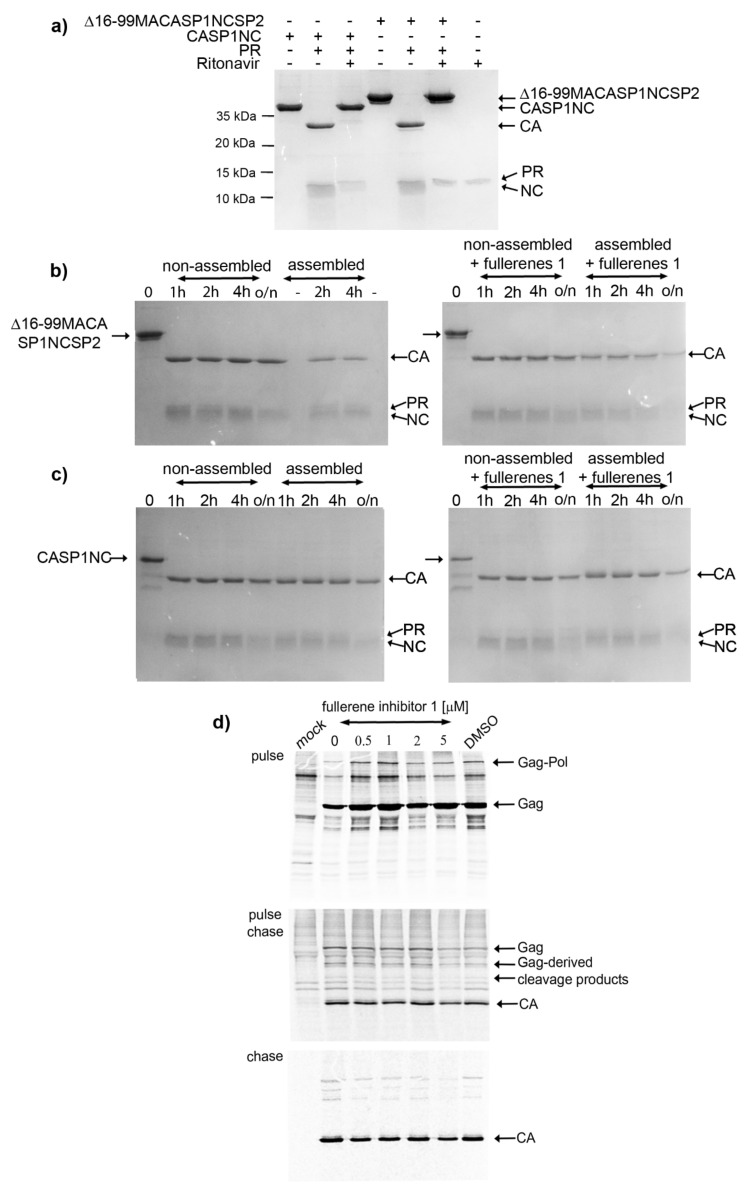
Effect of fullerene 1 on *in vitro* and virus-based maturation. (**a**) Western blot analysis of products of Δ16-99MA-CA-SP1-NC-SP2 and CA-SP1-NC cleavage by HIV-1 PR in the absence or presence of ritonavir. Western blot analysis of maturation of (**b**) MA-CA-SP1-NC-SP2 and (**c**) CA-SP1-NC mediated by HIV-1 PR in the absence or presence of 5 µM fullerene 1. (**d**) Expression (pulse), maturation, and release (pulse chase and chase) of HIV-1 Gag and Gag–Pol polyproteins. HEK 293 cells were pulse-labeled for 30 min with Tran35S-label and chased overnight in complete DMEM. Viral proteins from the cell lysates (pulse and pulse-chase) and media (chase) were immunoprecipitated by anti-HIV CA antibody. Proteins were separated by SDS-PAGE and detected by using a Typhoon PhosphorImager.

Castro et al., in their recent work, reported that fullerene derivatives affect the processing of HIV-1 Gag, but not Pol, polyprotein precursors [36]. Their data showed that the fullerene compounds blocked Gag processing at different cleavage sites, resulting in improperly cleaved Gag-derived proteins, including MA-CA-SP1-NC, MA-CA, and CA-SP1-NC. However, apart from the fuzzy-like appearance of the bands corresponding to CA, our immunoanalysis of HIV-1 Gag processing did not reveal any Gag-derived cleavage intermediates (Figure 1d,e). To clarify a potential effect of fullerene **1** on HIV-1 Gag maturation, we proceeded to a maturation study. First, we confirmed the specificity and activity of recombinant HIV-1 protease, produced and purified as described earlier [51]. The Gag-derived polyproteins Δ16-99MA-CA-SP1-NC-SP2 and CA-SP1-NC were incubated with HIV-1 protease, in the presence of the protease inhibitor ritonavir (Figure 2a). As expected, no cleavage of Gag polyprotein was observed in the sample containing ritonavir, whereas two main cleavage products—CA and NC proteins—were detected following the incubation with HIV-1 PR in the absence of this inhibitor (Figure 2a). To determine whether fullerene **1** inhibits the processing of HIV-1 Gag polyprotein, we analyzed both assembled and non-assembled forms of truncated Gag proteins. Non-assembled HIV-1 Gag-derived polyproteins (i.e., Δ16-99MA-CA-SP1-NC-SP2 and CA-SP1-NC) or *in vitro* prepared immature-like and mature-like particles assembled from the same proteins in the presence of **1** were incubated with HIV-1 PR. The effect of **1** on maturation was analyzed by comparison of Coomassie-stained proteolytic products resolved by SDS-PAGE at the indicated times (Figure 2b,c). No effect of fullerene **1** on the cleavage of Δ16-99MA-CA-SP1-NC-SP2 or CA-SP1-NC by HIV-1 protease was observed. Both assembled and free HIV-1 Gag-derived polyproteins were cleaved into two major proteins: CA and NC (Figure 2b,c).

To confirm the *in vitro* maturation results, we performed a pulse-chase experiment, during which the proteins produced in HEK 293 cells were metabolically labelled with ^35^S and monitored for different time periods; and virus-derived proteins were immunopecipitated (Figure 2d, pulse). HIV-1 Gag and Gag–Pol polyproteins were produced in the presence of various amounts of fullerene **1** (from 0.5–5 µM). No effect of fullerene **1** on the amount of HIV-1 Gag or Gag–Pol produced was observed (Figure 2d, pulse). Proper Gag maturation to expected Gag-derived cleavage products was detected after 2 h (Figure 2d pulse-chase). Properly processed CA, a major HIV-1 structural protein, was observed in the absence and in the presence of an increasing concentration of fullerene **1** during 24 h (Figure 2d chase). The relative release of HIV-1 particles was determined from the pulse-chase experiments by calculation of cell- and virus-associated protein levels (Figure 2d). The results of the pulse-chase, together with the *in vitro* maturation experiments, confirmed that fullerene **1** had no significant impact on the proper maturation and processing of HIV-1 polyprotein precursors or virus release.

### 3.3. Effect of 1 on the Assembly of HIV Immature and Mature Particles

**Figure 3:**
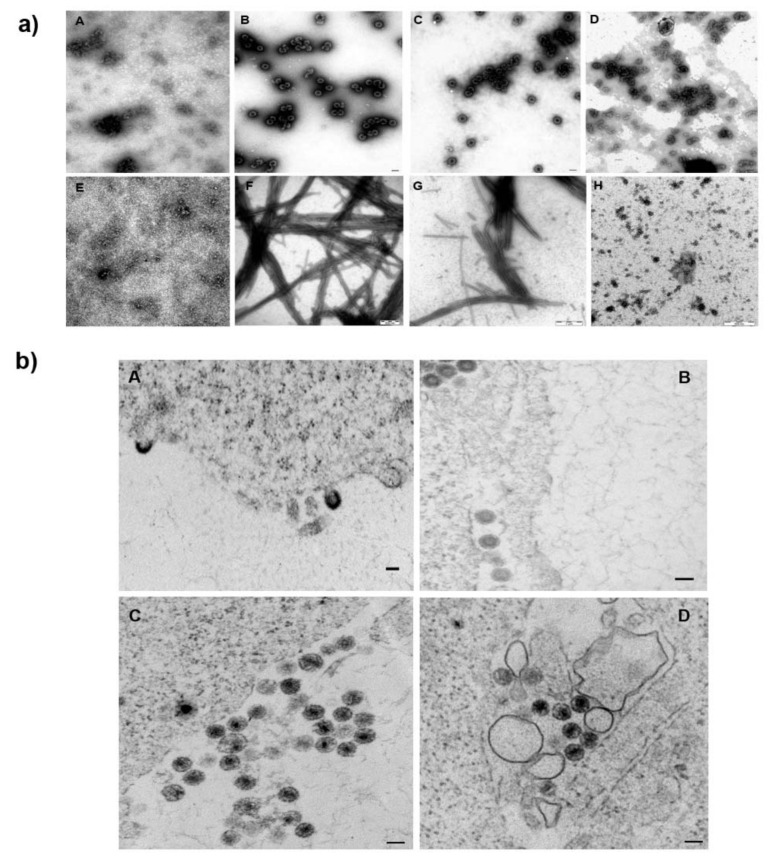
Effect of fullerene 1 on the assembly of immature and mature HIV-1 particles. (**a**) Representative TEM pictures of negatively stained particles assembled from (panels (**A**–**D**)) Δ16-99MACANCSP2 and (panels (**E**–**H**)) CANC in the absence of tqON (panels (**A**,**E**)), in the presence of tqON (panels (**B**,**F**)), in the presence of fullerene **1** (panels (**C**,**G**)), and in the presence of assembly inhibitor CAI (panels (**D**,**H**)). (**b**) TEM analysis of HEK 293 cell producing VSV-G-HIV-1 in the absence (**A**,**C**) or presence of fullerene **1** (**B**,**D**). Bars represent 100 nm.

It was postulated that the interaction of fullerene derivatives with HIV-1 polyproteins within the virus-producing HEK293 cells could affect CA-SP1’s maturation by modification of the Gag conformation, potentially compromising its ability to assemble [35]. To verify this hypothesis, we first employed an *in vitro* fast assembly inhibitor test for HIV FAITH [20,39,52,53] to analyze whether **1** had any effect on the assembly of virus-like particles (VLPs). This assay was established to quantify the efficiency of assembly of both HIV-1 immature-like spherical particles assembled from Gag polyprotein, carrying a deletion within the *N*-terminal matrix domain and lacking the C-terminal p6 domain—Δ16-99MACASP1NCSP2—and mature-like, tubular particles assembled from CASP1NC. The assembly is triggered by the addition of a short (40-mer), TaqMan-like oligonucleotide (tqON), which binds to the NC domain and is encapsulated into the assembling particle. In the presence of an inhibitor, the HIV-1 particles are not assembled, and tqON remains free in solution, not protected within the particle. When Exonuclease I is added to the reaction mixture, it degrades the free, unincorporated tqON. During tqON degradation, Exonuclease I separates the fluorophore from its quencher at tqON and the fluorescence is measured with a spectrofluorometer. The efficiency of the assembly is then calculated as explained in the Section 2. To determine the impact of **1** on the assembly efficiency, we mixed the HIV Gag-derived polyproteins Δ16-99MACASP1NCSP2 and CASP1NC with either 1 or a well-known peptide assembly inhibitor CAI, used as a positive control [54]. However, due to unexpected repeatedly observing a reduction in fluorescence in the control sample containing only tqON and **1**, we could not quantify the effect of 1 on the HIV-1 assembly. Therefore, only TEM analysis of negatively stained Δ16-99MACASP1NCSP2 and CASP1NC samples assembled in the presence and absence of 1 was used. In the absence of tqON, non-assembled material consisting mainly of free proteins was observed (Figure 3a, panels A, E). In contrast when tqON was present, spherical immature-like or tubular mature-like particles were observed for Δ16-99MACASP1NCSP2 (Figure 3a, panel B) and CASP1NC (Figure 3a, panel F), respectively. No morphological changes in immature-like or mature-like HIV particles were observed in the presence of **1** by TEM (Figure 3a, panels C, G). As expected, no particles were formed in the presence of CAI (Figure 3a, panels D, H).

We then verified that **1** does not affect the assembly of HIV-1 particles produced in HEK 293 cells. Thin sections of HEK-293 cells producing HIV-1 in the absence (Figure 3b, panels A, C) or presence of **1** (Figure 3b, panels B, D) were prepared and analyzed. No visible impact of 1 on the morphology of both immature (Figure 3b, panels A, B) and mature (Figure 3b, panels C, D) HIV particles was observed.

### 3.4. Effects on the Uncoating and Reverse Transcription

**Figure 4:**
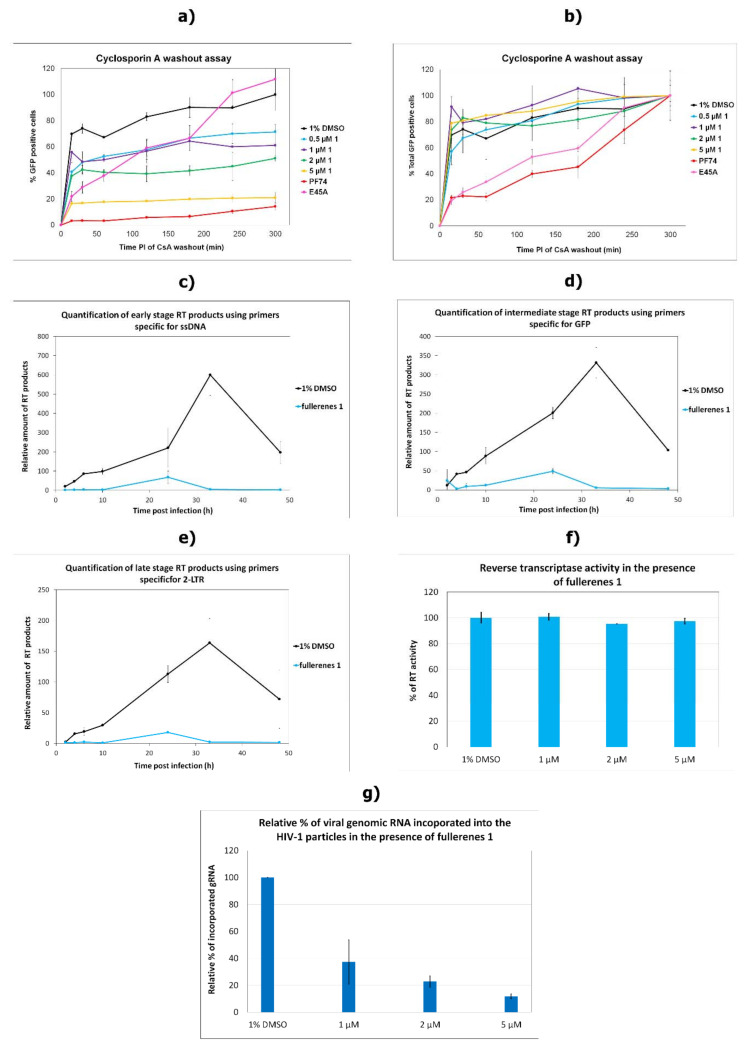
The effects of fullerene **1** on the selected steps of early phase of HIV-1 replication cycle—HIV-1 uncoating. (**a**,**b**) CsA washout assay. OMK cells were infected by normalized amounts of VSV-G pseudotyped HIV-1 produced in the presence of indicated concentration of fullerene **1**, or 5 µM PF74 was added. At the indicated times post-infection, the CsA-containing medium was replaced with fresh, CsA-free culture medium. The percentage of GFP-positive OMK cells was determined by flow cytometry and (**a**) normalized to the number of DMSO-containing non-drug control cells, which was considered 100%, or (**b**) normalized by setting the number of GFP-positive cells to 100% for HIV-1 reverse transcription. Progress of reverse transcription, (**c**) early stage, (**d**) intermediate stage, and (**e**) late stage. VSV-G pseudotyped HIV-1 particles produced in the absence or presence of 5 µM fullerene **1** were used to infect fresh HEK 293 cells, which were harvested 2, 4, 6, 10, 24, 33, and 48 h post-infection. Real time PCR analysis of isolated DNA was used to detect different reverse transcription products. The results were normalized for CA content using semi-quantitative Western blotting and two housekeeping genes: phospholipase A and C-C chemokine receptor type 5—CCR5. (**f**) Activity of reverse transcriptase was measured using a reverse transcriptase assay, colorimetric. Released virions produced in HEK 293 cells in the presence of indicated concentrations of fullerene **1** were harvested and centrifuged 48 h post-transfection. The subsequent steps were performed according to the manufacturer’s protocol (Merck). (**g**) Amount of viral gRNA incorporated into HIV-1 particles. The amount of gRNA from HIV-1 particles released from HEK 293 cells treated by DMSO or the indicated amount of **1** was quantified by real-time PCR analysis. If not stated otherwise, results represent two independent experiments.

As none of the steps of the late phase examined in our study were affected by **1**, we focused on the processes in the early stages of the HIV-1 replication cycle. Upon entry of the viral core into the new host cell, the uncoating is initiated. To study whether **1** could affect HIV-1 core uncoating, we employed a cyclosporine (CsA) washout assay [12,39,46]. This assay is based on the findings that the HIV-1 restriction factor TRIM5α-cyclophilin A (CypA) binds to the CA hexameric lattice of the mature HIV-1 core and blocks the uncoating. In the presence of CsA, the HIV-1 sensitivity to the restriction factor is lost. In the CsA washout assay, CsA is gradually washed out of the cells, enabling TRIM5α-CypA to bind to the CA lattice and accelerate HIV-1 uncoating. The presence of CsA does not rescue HIV-1 with impaired uncoating, and the infectivity is blocked. To determine whether **1** affected HIV-1 uncoating, we produced VSV-G pseudotyped GFP-HIV-1 virions in the presence of various concentrations of **1**. Semi-quantitative Western blot-normalized amounts of these virions were used to infect the owl monkey kidney cells (OMK) endogenously expressing TRIM5α-cyclophilin. Uncoating inhibitor PF74 and CA E45A mutant, which was shown to stabilize mature core and delays the uncoating [55], were used as experimental controls. Following CsA washouts at different time intervals, shown in Figure 4a, the percentages of GFP-positive cells (i.e., infected cells) were determined by flow cytometry. The obtained results were normalized either to the DMSO-treated cells by setting the percentage at 5 h as 100%, as shown in Figure 4a, or by setting the percentage of GFP-positive cells of all samples at 5 h post transfection to 100% (Figure 4b). A clear impact of **1** on HIV-1’s infectivity toward OMK cells was observed (infectivity was reduced; see Figure 4a). In agreement with the published data [56], our results on CsA washout assay showed that both E45A CA HIV-1 and PF74 delay significantly the HIV-1 uncoating (Figure 4b). In contrast, no such delay was observed for HIV-1 in the presence of fullerene **1**. The results of the CsA washout assay suggest that **1** did not delay the HIV-1 core uncoating, or in other words, that **1** most probably had no effect on the mature hexameric lattice stability.

The uncoating is tightly connected with the reverse transcription. Martinez et al. showed that while the packaging of HIV-1 genomic RNA (gRNA) into the virus was not affected by C_60_ fullerenes, a dramatic reduction in viral cDNA synthesis occurred [35]; however, this characterization was not done for C_70_ derivatives. As HIV-1 cDNA is formed only during reverse transcription, we tested its synthesis by using quantitative PCR (qPCR) of DNA isolated at various times post-infection with HIV-1 produced in the presence of **1** (5 µM). As previously reported [35], we also observed severe blocking in all stages of HIV-1 cDNA synthesis during reverse transcription in the presence of **1** (Figure 4c–e). To determine whether **1** could interfere with RT activity, as was reported for C_60_ fullerenes [35], we purified HIV-1 particles produced in HEK 293 cells treated with various concentrations of **1** and measured RT activity using a colorimetric assay, as described earlier [45]. In contrast to C_60_ fullerenes [35], we detected very similar levels of RT activity in the absence and in the presence of various concentrations of **1** (Figure 4f). These data indicated that despite the pronounced effect of C_60_ and C_70_ fullerenes on the yield of reverse transcription, the activity of RT remained unimpaired in the presence of C_70_ fullerenes. Next, we compared the amounts of viral gRNA present in the virions produced in the presence and absence of fullerene **1.** Viral gRNA was isolated from the semi-quantitative Western blot-normalized HIV-1 particles, and the level of incorporated gRNA was determined using qPCR. The amount of incorporated gRNA was decreasing in a fullerene **1** dose-dependent manner (Figure 4e). These data suggest that **1** interferes with packaging of viral genomic RNA into nascent viral particles.

### 3.5. In Vitro Binding Affinity of Fullerene 1 to HIV-1 Proteins

To investigate whether the impaired gRNA incorporation resulting in blocked reverse transcription is connected to direct binding of fullerene **1** to CA, NC, or nucleic acid, micro-scale thermophoresis (MST) was used. HIV-1 PR, which was shown to interact with fullerene derivatives, was also used [33,34].

**Figure 5:**
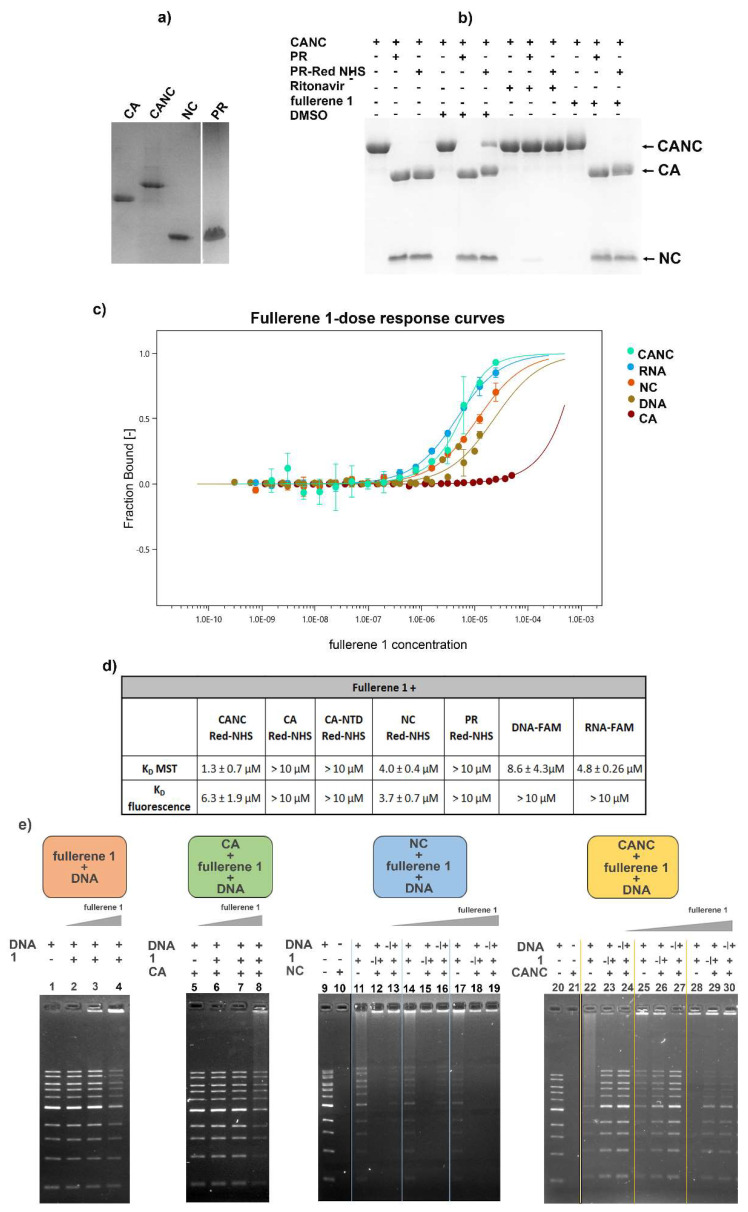
Interactions of fullerene **1** with HIV-1 CANC, CA, NC, and PR analyzed by microscale thermophoresis measurement (MST) and electrophoretic mobility shift assay (EMSA). (**a**) SDS PAGE of HIV-1 proteins used in MST and EMSA analysis. (**b**) HIV-1 PR following THS-RED labeling was tested for its proteolytic activity using the indicated samples. (**c**) Dose–response plot of different concentrations of fullerene **1** after the interaction with fluorescently labeled HIV-1 proteins and nucleic acids calculated from MST analysis. Error bars represent standard deviations. (**d**) Dissociation constants (K_D_) calculated from MST for fullerene **1** and indicated HIV-1 proteins. (**e**) EMSA: the same amounts of DNA (200 ng) and HIV-1 protein (1 μM) were used in all samples. The concentrations of **1** (1, 2, and 5 μM) at the final molar ratios of protein:fullerene **1** corresponding to 1:1, 1:2, and 1:5 were used. Orange panel: DNA was incubated with DMSO (1%) (lane 1) or with various amounts of **1**, corresponding to 1, 2, and 5 μM in 1% DMSO (lanes 2–4); green panel: HIV-1 CA was incubated with DNA (lane 5) or with various amounts of **1**, corresponding to 1, 2, and 5 μM (lanes 6–8). To analyze the interactions among HIV-1 NC, **1**, and nucleic acid (blue panel), two experiments were performed. In one, NC was first incubated with DNA and then with **1** (1, 2, or 5 μM) at the final molar ratios 1:1, 1:2, and 1:5 (lanes 12, 15, 18 respectively). In the second, NC was first preincubated with the various amounts of **1**, and then incubated with DNA (lanes 13, 16, and19). As controls, NC was incubated with DMSO (1%) in the absence of **1** (lane 10), and DNA was incubated with the various amounts of **1** (lanes 11, 14, and 17). CANC’s interactions with **1** and DNA (yellow panel) were tested identically as described for NC: CANC was preincubated with DNA, and then with **1** (lanes 23, 26, and 29), or CANC was preincubated with various amounts of **1**, and then incubated with DNA (lanes 24, 27, and 30). DNA binding to various amounts of **1** was also analyzed (22, 25, and 28). All samples were incubated for 40 min at RT and then analyzed using 0.8% agarose gel electrophoresis, stained with Gel Red, and visualized with a Quantum gel documentation imaging system (Vilbert Lourmat).

For the MST, the analyzed HIV-1 proteins—CA, NC, CANC, and PR (Figure 5a)—were prepared and fluorescently labelled using Protein Labeling Kit Red-NHS (Nanotemper Technologies). The proper dimer formation of fluorescently labeled HIV-1 PR was confirmed by its proteolytic activity on CA-NC (Figure 5b). FAM-labeled RNA and DNA oligonucleotides were purchased from Merck Life Sciences. During the MST measurements, we kept all labeled samples at constant concentrations, and the final concentration of **1** ranged from 15 nM to 50 µM (Figure 5c). The determined K_D_ values of fullerene **1** to selected HIV-1 proteins are summarized in Figure 5d. The strongest interaction, corresponding to K_D_ 1.3 µM, was observed for **1** and non-assembled HIV-1 CANC. Lower binding affinity (K_D_ 4 µM), though still comparable with that of **1** to CANC, was determined for mature HIV-1 NC. Fullerene **1** revealed also binding affinity for both RNA and DNA oligonucleotides. RNA oligonucleotides showed slightly higher affinity towards the fullerene; however, the K_D_ was about three times a higher compared to that of **1** and CANC (4.8 µM for RNA, 8.6 µM for DNA). K_D_ values for full-length CA or its N-terminal domain, and for PR, were at least one-order of magnitude higher compared to those for CA-NC and NC. However, due to the low solubility of **1**, we were not able to determine exact K_D_s at higher concentrations.

To verify the affinities of **1** for CA, NC, CANC, and nucleic acid (ssDNA), we performed EMSA analysis (Figure 5e). The idea of this experiment was as follows: if fullerene **1** binds nucleic acid, the binding should be concentration-dependent. If **1** binds with a higher affinity for the protein than to DNA, the protein should prevent the fullerene’s binding to DNA, which should remain free, not forming a complex. If **1** does not bind to the protein, the fullerene–DNA binding profile should not be affected by the addition of the protein. Fullerene **1** indeed bound ssDNA in a concentration-dependent manner (Figure 5e), and it formed a complex that did not enter the agarose gel and remained at the top in the loading well (Figure 5e, lanes 3, 4, 11, 14, 17, 22, 25, and 28). When HIV-1 CA was mixed with DNA in the absence of **1**, no complex was formed (Figure 5e, green panel, lane 5) which is in agreement with the fact that the CA protein has no strong nucleic acid binding capacity. When **1** was added to CA and then DNA was added, the same binding profile as for DNA and **1** was observed, suggesting that majority of fullerene **1** remained unbound by CA and available for binding to DNA (Figure 5e—compare lanes 2–4 of the orange panel and 6–8 of the green panel). As NC naturally and efficiently binds DNA, the analysis of the interactions of NC, CANC, **1,** and DNA is complicated. To analyze whether binding of **1** to NC can interfere with NC’s affinity for DNA, two experiments were performed: In the first one, HIV-1 NC or CANC was incubated with an excessive amount of DNA, following by the addition of **1** (various amounts)**.** After incubation, the NC and CANC-containing samples were resolved by 0.8% agarose gel electrophoresis (Figure 5e, blue panel, lanes 12, 15, and 18; and yellow panel lanes 23, 26, and 29, respectively). In the second experiment, NC and CANC proteins were first pre-incubated with various amounts of **1**, and then DNA was added. The comparison between these two shows that pre-incubation of **1** with NC and CANC prevented the NC moiety from binding to the nucleic acid, as more free DNA remained in the gel (compare the lanes 12/13 and 15/16 for NC; and 23/24 and 26/27 for CANC in Figure 5e). Together with MST analysis, these data strongly suggested that fullerene **1** preferentially bound the NC domain of HIV-1 Gag, blocking its interaction with nucleic acid mimicking viral genomic RNA.

In conclusion, we showed that fullerene **1** blocked HIV-1 infectivity by reducing the amount of viral gRNA packed into HIV-1 particles depending on the concentration of inhibitor. The combination of the MST and EMSA analysis data strongly argues that although fullerene **1** can bind both NC and nucleic acid, it preferentially binds the NC domain of HIV-1 Gag, thereby blocking its interaction with viral genomic RNA.

## 4. Discussion

In this work, we studied the mechanism of the anti-HIV-1 activity of *N*,*N*-dimethyl[70]fulleropyrrolidinium iodide fullerene derivative **1** by investigating various steps of the HIV-1 replication cycle: assembly, release, processing and maturation, viral genomic RNA incorporation, uncoating, and reverse transcription. We found that **1** affected the amount of viral genomic RNA in HIV-1 viral particles in a concentration-dependent manner. Logically, we also studied the impact of **1** on the processes associated with reverse transcription; however, neither reverse transcriptase nor protease was inhibited by these C_70_ derivatives. Using MST, we observed a strong binding affinity of fullerene **1** for HIV-1 NC. This binding was observed for both forms of NC, the Gag-mimicking immature domain (within the CA-NC precursor) and the mature NC protein. Fullerene **1** also bound nucleic acids; however, its affinity for the immature form of NC (CA-NC fusion protein) was about three times higher than that to RNA oligonucleotide. In contrast, the affinities of **1** for CA protein and HIV-1 protease were at least one order of magnitude lower. Altogether, our results suggest that fullerene **1** binds to HIV-1 NC and thus affects its ability to specifically interact with viral genomic RNA, thereby blocking HIV-1 infectivity.

Previously it was suggested that fullerene derivatives affect HIV-1 maturation [35,36]. One of the reasons why **1** was suggested to act as a maturation inhibitor was the presence of partially processed CA-containing Gag fragments such as CA-SP1-NC and CA-SP1 in fullerene-treated HEK293 cells producing HIV-1 Gag [35,36]. However, a more detailed analysis performed here via both *in vitro* and cell-based HIV-1 Gag polyprotein processing analysis with a C_70_ fullerene-derivative excluded this mechanism of action. We did not observe any impact on HIV-1 protease activity or Gag polypeptide processing under *in vitro* conditions, where recombinant HIV-1 protease was added to Gag-derived, purified polypeptides Δ16-99MACASP1NCSP2 and CASP1NC. There was a possibility that the fullerene-dependent effect on HIV-1 processing could be connected to conformational changes of Gag polyprotein occurring during assembly. However, we found no impact of **1** on the processing of *in vitro* assembled spherical, immature-like Δ16-99MACASP1NCSP2 or tubular, mature-like CASP1NC particles. To verify this *in vitro* observation, we analyzed the effect of **1** on the processing and maturation of HIV-1 Gag and Gag–Pol polyproteins authentically produced in HEK 293 cells. Similarly, viruses released from fullerene-derivatives treated HEK 293 cells showed no defects in Gag processing. At all tested concentrations (0.5–5 µM) of **1**, we observed the HIV-1 polyprotein precursors (i.e., Gag Pr55 and Gag–Pol 160) and mature CAp24 of expected sizes and in ratios comparable to those of the fullerene non-treated HIV-1 producing cells.

The analysis of the cells from the chase experiment showed both fully processed CA and partially processed CA-containing Gag fragments. However, both these forms were present also in the non-treated or DMSO-only treated control samples. We assume that these CA-containing Gag fragments are generated during the initial phase of maturation when the budding viral particles still remain attached to the membrane. This assumption is supported by the pulse-chase experiment where the viruses released in the culture media contained fully processed CAp24 at all fullerene concentrations tested. The fuzzy-like appearance of the CAp24 band, which we also observed by Western blot analysis (Figure 1d,e), was, however, not observed in the pulse-chase experiments. This may have been due to different sample treatment in the pulse-chase (i.e., immunoprecipitation) compared to the Western blot. Nevertheless, based on the above discussed results, we conclude that **1** did not affect HIV-1 Gag polyprotein maturation.

In their work, Castro et al. [36] suggested a direct interaction between the fullerene inhibitor and HIV-1 capsid protein. As CA is the major structural protein critical for the formation of HIV-1’s immature and mature hexameric lattices and for uncoating, we next tested the possible direct binding of **1** to CA. We did not observe any effect of **1** on the HIV-1 assembly either by using an *in vitro* assembly system or by TEM analysis of the thin sections of the HEK 293 cells producing HIV-1. No effect of **1** was detected in HIV-1 CA uncoating. The CsA washout assay, which monitors the HIV-1 capsid core uncoating, clearly showed a concentration-dependent effect of **1** on virus infectivity in OMK cells. However, the rate of uncoating, which would prove an effect of **1** on hexameric CA lattice’s core stability, was not affected by fullerene **1**. In comparison to PF74 and E45A CA mutant, which were both shown to affect capsid hexameric stability [39,55,57,58,59], fullerene **1** did not slow down the rate of uncoating. Finally, as MST analysis confirmed the low-binding affinity of **1** to HIV-1 CA, we concluded that **1** did not directly bind to HIV-1 CA.

Since the rate of uncoating seemed to be unaffected by fullerene **1**, to understand its effect on reverse transcription, we studied the direct effect of fullerene **1** on RT activity. No inhibitory effect was observed, however, so we quantified the amount of viral genomic RNA incorporated into released HIV-1. Surprisingly, we found an inverse correlation between the concentration of **1** and the amount of viral genomic RNA. That was unexpected, because Martinez et al. did not observe any effect of fullerene inhibitors on RNA incorporation [35]. In their work they normalized fullerene derivatives-treated virions by ELISA. However, we noticed that fullerene strongly interfered with the horseradish peroxidase (HRP) conversion of the 3,3′,5,5′-tetramethylbenzidine (TMB) substrate to its oxidized colored product (Supplementary Figure S1a). Therefore, **1** might severely skew the ELISA results and subsequent experiments based on ELISA-normalized virions (such as virus-associated genomic RNA). To avoid this fullerene-associated bias, we used a semi-quantitative Western blot for virions normalization [40]. Apart from **1**’s interference with the TMB reaction, we also observed that during MST analysis, **1** quenched the fluorescence of its fluorescently labeled interacting partner (e.g., NHS-RED-labeled CANC). We saw no quenching of FAM or NHS-RED dye itself (Supplementary Figure S1b). This observation clarified the problems connected to FAITH measurements, during which we observed fullerene **1** concentration-dependent fluorescence quenching of tqON (Supplementary Figure S1c). This fluorescent quenching property of **1** could explain the discrepancies between our results and Martinez et al.’s results on the effect of **1** on HIV-1 RT activity [35]. Using the assay with the chromogenic substrate (Merck), which is not affected by fullerene **1,** we did not observe any impact of **1** on RT activity (Figure 4g). In contrast, when Martinez et al. used the same assay, the formation of fluorescently labeled RNA–DNA heteroduplex was detected. As we showed by MST that **1** binds to FAM-labeled RNA or DNA and quenches their fluorescence, it is plausible that binding of **1** to fluorescently labeled nucleic acid decreased its fluorescence, which was then misinterpreted as reduced RT activity.

**Figure 6:**
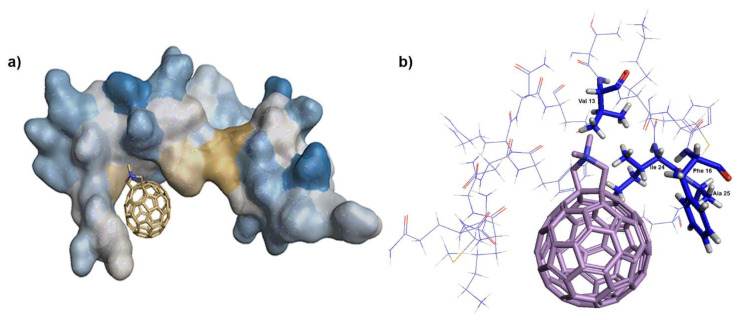
Docking of fullerene **1** into HIV-1 NC. Docking into NC (PDB 1F6U) was carried out in two programs, PLANTS and AutoDock Vina: (**a**) Heat map of NC with docked fullerene **1** (blue: hydrophilic, gold: hydrophobic). (**b**) A detailed illustration of fullerene **1** and the NC hydrophobic plateau formed by Val13, Phe16, Ile24, and Ala25 of the NC proximal ZF (the hydrophobic residues are drawn in capped sticks and marked by labels).

The logical explanation for how fullerene **1** affected viral genomic RNA incorporation could be its interference with NC–gRNA binding. We proved the affinity of **1** to CANC and NC by MST. CANC–fullerene and NC–fullerene **1** binding were also verified by EMSA, during which NC treated by **1** reduced its ability to interact with ssDNA in an inhibitor concentration-dependent manner. Although we did not observe the direct binding of HIV-1 CA to fullerene using MST or ELISA assays, in two experiments (Figure 1d and Figure 5b), a fuzzy-like band, supposedly corresponding to CA with bound fullerene **1**, was observed. A similar fuzzy-like appearance of the HIV-1 CA band, as a result of fullerene inhibitor binding, was previously identified by Castro et al. [36]. They performed a pull-down analysis by incubating fullerene inhibitors attached to magnetic beads with the lysate of HEK-293 cells producing HIV-1 Gag and Pol polyproteins [36]. Based on the fuzzy-like appearance of this immunoprecipitated CA on the immunoblot, Castro et al. concluded that the molecular weight did not correspond to fully processed CAp24, but possibly to the CA-SP1p25 protein. The reason for these fuzzy bands in our Western blot (Figure 1d) and Coomassie-blue stained gel (Figure 5b) remains unclear. However, in contrast to bevirimat, we did not observe any effect of **1** on HIV-1 CA maturation by using immunoprecipitation of radioactively labelled HIV-1 Gag derived proteins (pulse-chase experiment). We therefore concluded that the fuzzy-like appearance is a technical artefact; nevertheless, it represented properly processed CAp24 protein. To confirm high preference of fullerene **1** in binding NC prior to CA, we performed docking experiments. We used two programs, PLANTS [48] and AutoDock Vina, version 1.5.6rc3 [49], to dock **1** into HIV-1 CA (3NTE) and NC (1F6U). In the case of CA, no preferential binding site was found, as both programs selected two different preferential binding sites, always in the ratio 80:20 (Supplementary Figure S2). In contrast, both programs placed **1** into the same position within the NC structure—into the vicinity of the hydrophobic residues Val13, Phe16, Ile24, and Ala25 of the proximal zinc finger (ZF) (Figure 6a,b). Together with the hydrophobic residues of the distal ZF, Trp37, Gln45, and Met46, these amino acid residues form a hydrophobic plateau, which plays a key role in NC binding to nucleic acids [18,60,61]. Even though we cannot completely rule out that fullerene **1** may also bind to HIV-1 CA hydrophobic pockets, our MST and EMSA data strongly argue that this interaction is insignificant and that fullerene **1** potently blocks infection by interfering in NC–gRNA interactions. The lowered amount of virus-incorporated gRNA then leads to a decrease in cDNA synthesis during reverse transcription and a significant reduction in HIV-1 infectivity.

## Data Availability

The data that support the findings of this study are available from the corresponding author upon reasonable request.

## Supplementary Materials

The following are available online at https://www.mdpi.com/1999-4915/13/12/2451/s1, Figure S1: Interference of fullerene **1** with ELISA, MST and FAITH, Figure S2: Docking of fullerene 1 into HIV-1 CA structure.
