# Correction: Křížová et al. Fullerene Derivatives Prevent Packaging of Viral Genomic RNA into HIV-1 Particles by Binding Nucleocapsid Protein. *Viruses* 2021, *13*, 2451

**DOI:** 10.3390/v14061187

**Published:** 2022-05-30

**Authors:** Ivana Křížová, Alžběta Dostálková, Edison Castro, Jan Prchal, Romana Hadravová, Filip Kaufman, Richard Hrabal, Tomáš Ruml, Manuel Llano, Luis Echegoyen, Michaela Rumlová

**Affiliations:** 1 Department of Biotechnology, University of Chemistry and Technology Prague, Technická 5, 166 28 Prague, Czech Republic; krizovaa@vscht.cz (I.K.); dostalkl@vscht.cz (A.D.); romana.hadravova@uochb.cas.cz (R.H.); kaufmanf@vscht.cz (F.K.); 2 Department of Chemistry, University of Texas at El Paso, 500 West University, El Paso, TX 79902, USA; edisoncastro2004@hotmail.com (E.C.); echegoyen@utep.edu (L.E.); 3 Laboratory of NMR Spectroscopy, University of Chemistry and Technology, 166 28 Prague, Czech Republic; prchalj@vscht.cz (J.P.); hrabalr@vscht.cz (R.H.); 4 Department of Biochemistry and Microbiology, University of Chemistry and Technology Prague, Technická 5, 166 28 Prague, Czech Republic; rumlt@vscht.cz; 5 Department of Biological Sciences, University of Texas at El Paso, 500 West University, El Paso, TX 79902, USA; mllano@utep.edu

The authors wish to make the following corrections to this paper [1]:

**Error in Figure**

The replacement of panels d and e in Figure 1 is due to our finding that Bevirimat-derived inhibitor was used by mistake instead of Bevirimat. Figure 1, with corrected panels d and e, is listed below.

Additionally, the authors also wish to publish the corrected panel e of Figure 5. Checking the raw electrophoretic data used for the assembly of the panel e of Figure 5, we realized that we unintentionally inserted the wrong lanes (5–8). Figure 5 with corrected panel e appears below.

The authors apologize for any inconvenience caused and state that the scientific conclusions are unaffected. This correction was approved by the Academic Editor. The original publication has also been updated.

## Figures and Tables

**Figure 1:**
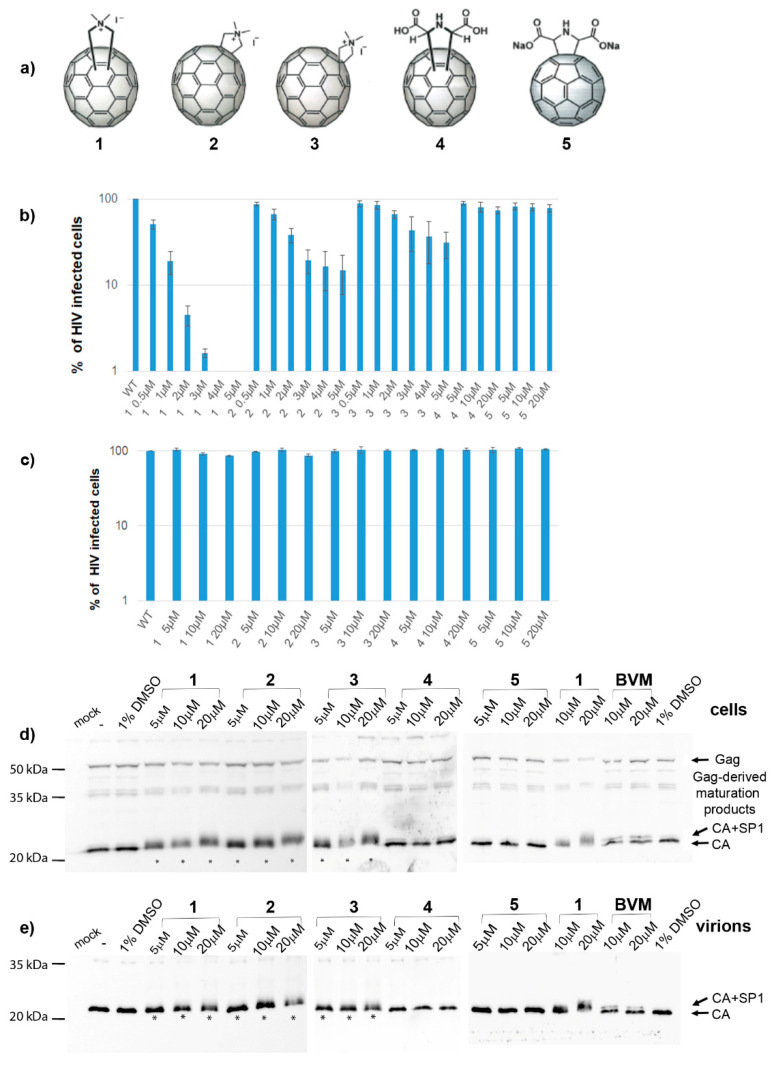
Fullerene derivatives and their effects on the HIV-1 life cycle. (**a**) Structures of fullerene derivatives 1-5. (**b**) HEK 293 cells produced VSV-G-pseudotyped GFP-HIV-1 in the presence of DMSO or fullerene derivatives at indicated concentrations. At 48 h post-transfection, a normalized amount of the VSV-G pseudotyped GFP-HIV-1 released into the culture media was used for infection of fresh HEK 293 cells, and 48 h later, the GFP-positive cells were quantified using a flow cytometer. (**c**) Effects of fullerenes on the early stage of HIV-1 life cycle. ELISA-normalized amounts of VSV-G pseudotyped GFP-HIV-1 viruses were used for infection of HEK 293 cells in the presence of DMSO or indicated concentrations of fullerene derivatives. Forty-eight hours later, the GFP-positive cells were quantified using a flow cytometer. (**d**) Immunoanalysis of intracellular VSV-G pseudotyped GFP-HIV-1 virus produced in HEK 293 cells in the presence of DMSO, fullerene derivatives, or bevirimat at the indicated concentrations. (**e**) Immunoanalysis of the VSV-G pseudotyped GFP-HIV-1 virus released from the HEK 293 cells.

**Figure 5:**
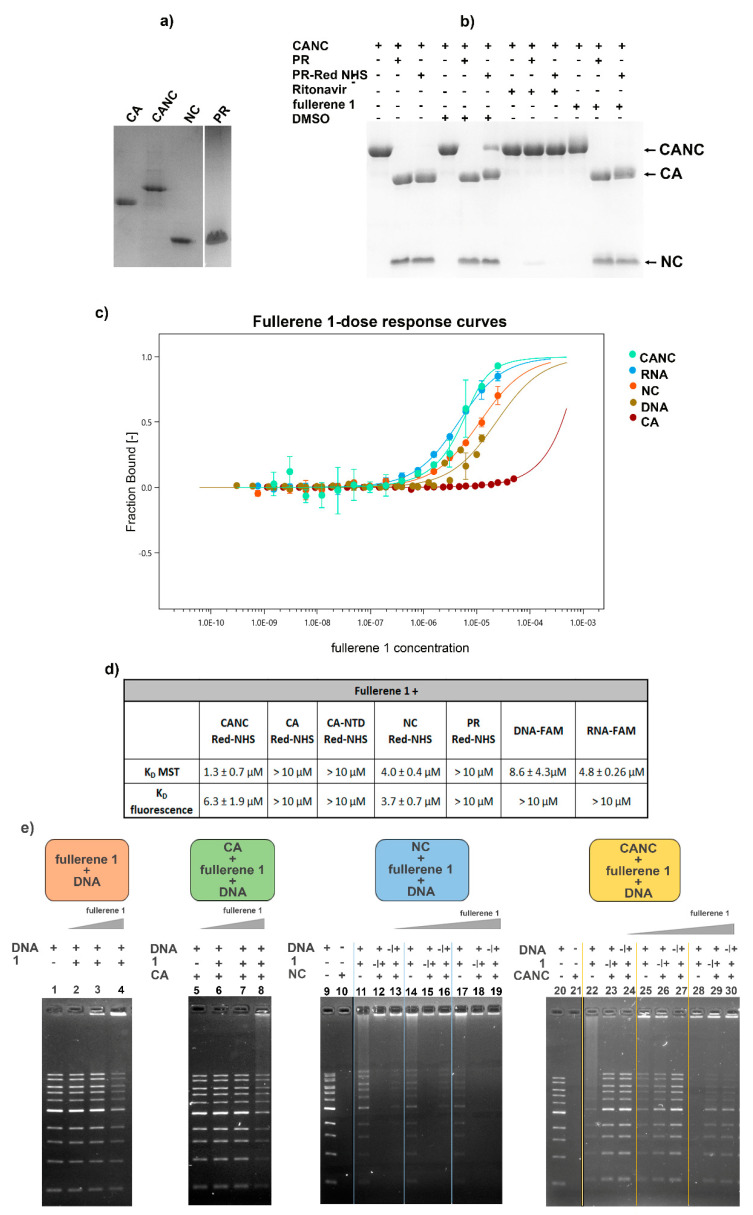
Interactions of fullerene 1 with HIV-1 CANC, CA, NC, and PR analyzed by microscale thermophoresis measurement (MST) and electrophoretic mobility shift assay (EMSA). (**a**) SDS PAGE of HIV-1 proteins used in MST and EMSA analysis. (**b**) HIV-1 PR following THS-RED labeling was tested for its proteolytic activity using the indicated samples. (**c**) Dose–response plot of different concentrations of fullerene 1 after the interaction with fluorescently labeled HIV-1 proteins and nucleic acids calculated from MST analysis. Error bars represent standard deviations. (**d**) Dissociation constants (K_D_) calculated from MST for fullerene 1 and indicated HIV-1 proteins. (**e**) EMSA: the same amounts of DNA (200 ng) and HIV-1 protein (1 μM) were used in all samples. The concentrations of **1** (1, 2, and 5 μM) at the final molar ratios of protein:fullerene 1 corresponding to 1:1, 1:2, and 1:5 were used. Orange panel: DNA was incubated with DMSO (1%) (lane 1) or with various amounts of **1**, corresponding to 1, 2, and 5 μM in 1% DMSO (lanes 2–4); green panel: HIV-1 CA was incubated with DNA (lane 5) or with various amounts of **1**, corresponding to 1, 2, and 5 μM (lanes 6–8). To analyze the interactions among HIV-1 NC, **1**, and nucleic acid (blue panel), two experiments were performed. In one, NC was first incubated with DNA and then with **1** (1, 2, or 5μM) at the final molar ratios 1:1, 1:2, and 1:5 (lanes 12, 15, 18 respectively). In the second, NC was first preincubated with the various amounts of **1**, and then incubated with DNA (lanes 13, 16, and 19). As controls, NC was incubated with DMSO (1%) in the absence of **1** (lane 10), and DNA was incubated with the various amounts of **1** (lanes 11, 14, and 17). CANC’s interactions with **1** and DNA (yellow panel) were tested identically as described for NC: CANC was preincubated with DNA, and then with **1** (lanes 23, 26, and 29), or CANC was preincubated with various amounts of **1**, and then incubated with DNA (lanes 24, 27, and 30). DNA binding to various amounts of **1** was also analyzed (22, 25, and 28). All samples were incubated for 40 min at RT and then analyzed using 0.8% agarose gel electrophoresis, stained with Gel Red, and visualized with a Quantum gel documentation imaging system (Vilbert Lourmat).
