# Supplementary material for: Fullerene Derivatives Prevent Packaging of Viral Genomic RNA into HIV-1 Particles by Binding Nucleocapsid Protein
Source: Viruses. 2021 Dec 6;13(12):2451. doi: 10.3390/v13122451 (PMC8705927; doi:10.3390/v13122451)

Fullerenes derivative prevents packaging of viral genomic RNA into HIV-1 particles due to binding nucleocapsid protein. Křížová I, et al.

Supplementary Figure S1. Interference of fullerenes **1** with ELISA, MST and FAITH. (**a**) Effect of **1** on ELISA signal: Fullerenes **1** at 1, 2 and 5µM or control sample containing only DMSO at final concentration 1% were incubated with anti-rabbit HRP-conjugated secondary antibody (Sigma-Aldrich) diluted 1:10,000 in reaction buffer were mixed with 100 µl of the TMB substrate (1 mg of 3,3′,5,5′-tetramethylbenzidine, Sigma-Aldrich diluted in 1 ml DMSO, 0.006% hydrogen peroxide, 0.05 M phosphate-citrate buffer, pH 5.0). The reaction was stopped by the addition of 50 µl of 2M sulfuric acid and the absorbance of the samples was measured at 450 nm using a Tecan M200 Pro. The HIV-1 CA amount was calculated using linear regression equation in the linear part of the calibration curve. (**b**) The effect of fullerenes 1 on MST signals of fluorescent dyes NHS-RED and FAM, and NHS-RED labeled HIV-1 CANC at increasing concentrations of 1 from 15 nM to 50 µM. The samples were loaded into Monolith NT.115 Capillaries (NanoTemper Technologies) and data of three independent measurements were analyzed using Affinity Analysis software version 2.3, (NanoTemper Technologies). (**c**) Dual-labelled tqON was pre-incubated with DMSO (of the final concentration 1%) or fullerenes 1 of the final concentration of 1, 2 and 5 µM for 1 h in 1% DMSO. Exonuclease I (ExoI) was added, and the fluorescence of the fluorophore released from degraded tqON was measured using a Tecan M200Pro plate reader.

a)

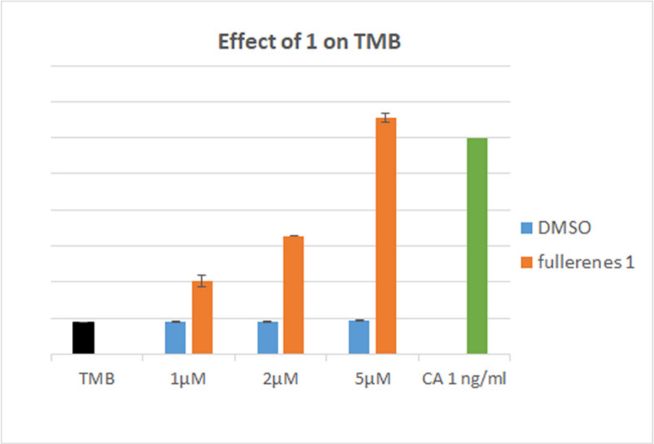
Effect of 1 on TMB
DMSO
fullerenes 1
TMB
1µM
2µM
5µM
CA 1 ng/ml

b)

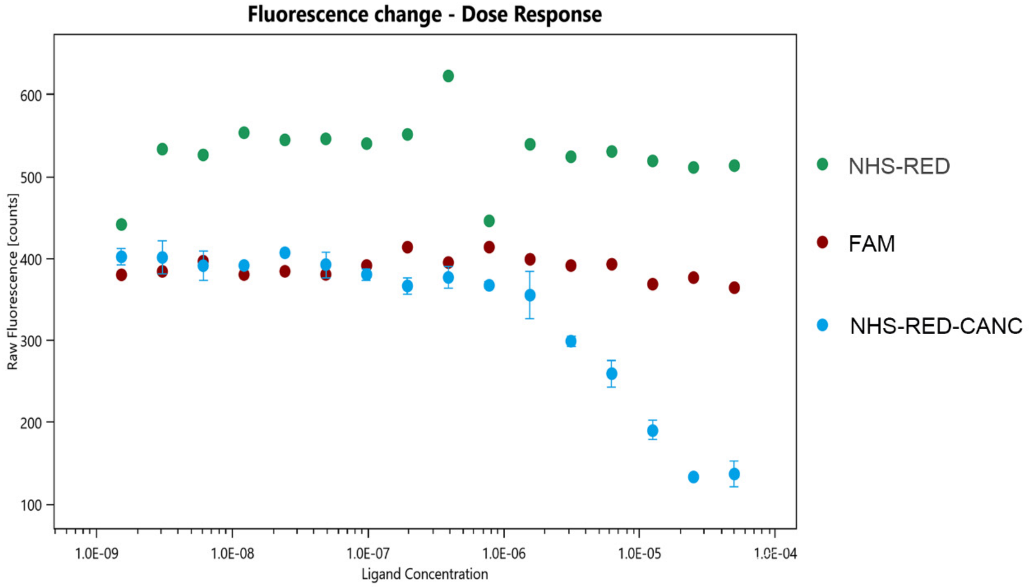
Fluorescence change - Dose Response
Raw Fluorescence [counts]
600
500
400
300
200
100
1.0E-09
1.0E-08
1.0E-07
1.0E-06
1.0E-05
1.0E-04
Ligand Concentration
NHS-RED
FAM
NHS-RED-CANC

c)

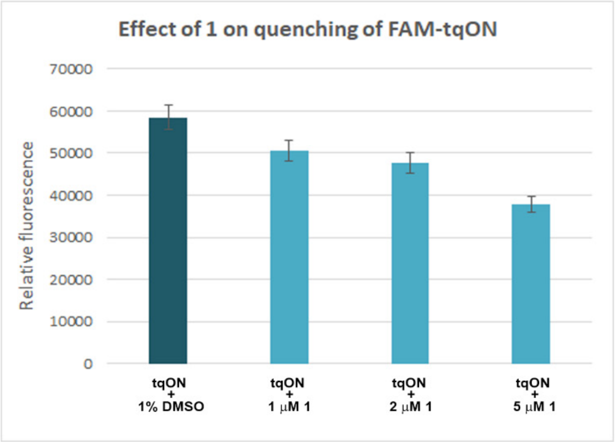
Effect of 1 on quenching of FAM-tqON
Relative fluorescence
70000
60000
50000
40000
30000
20000
10000
0
tqON + 1% DMSO
tqON + 1 µM 1
tqON + 2 µM 1
tqON + 5 µM 1

Supplementary Figure S2. Docking of fullerenes **1** into HIV-1 CA structure. Docking into CA structure (3NTE) was carried out in two programs, PLANTS and AutoDock Vina. PLANTS selected binding sites 1 (80%) and 3 (20%) whereas Autodock-Vina docked fullerenes 1 into 2 (80%) and 3 (20%).

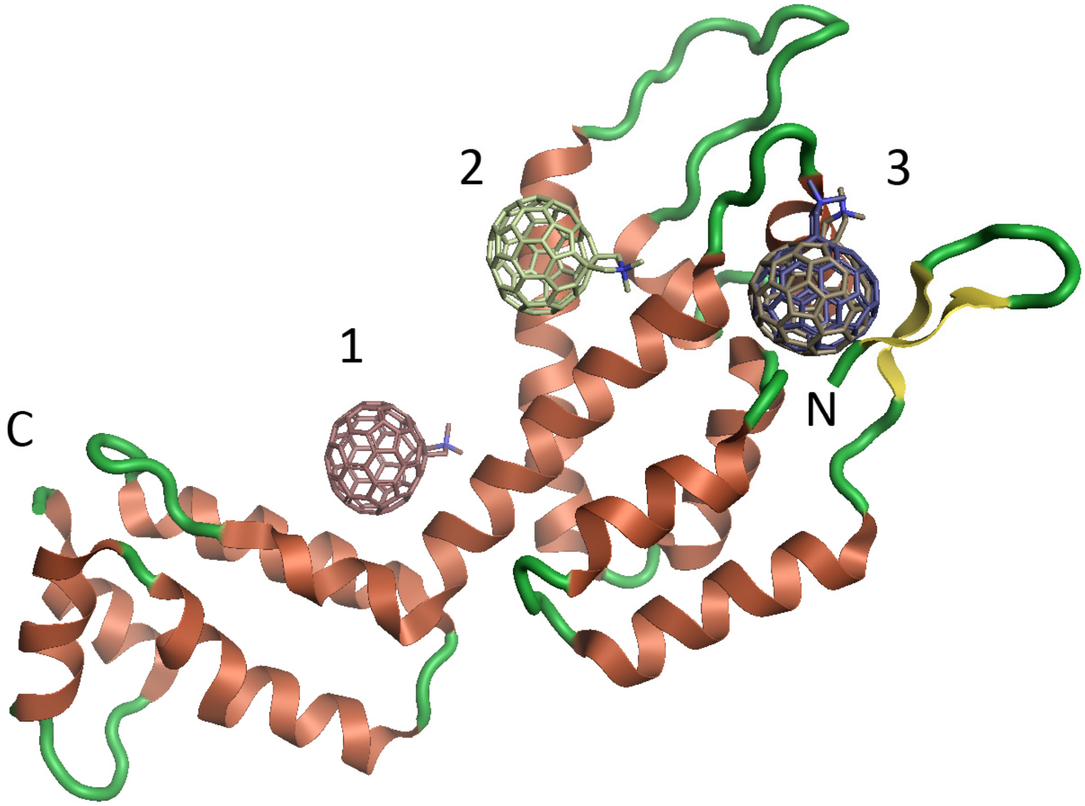

Supplement: Supplementary file 1 [file viruses-13-02451-s001.zip › Krizova et al. 2021 Supplementary Figures.pdf]
